# Improved Real-Time Fire Warning System Based on Advanced Technologies for Visually Impaired People

**DOI:** 10.3390/s22197305

**Published:** 2022-09-26

**Authors:** Akmalbek Bobomirzaevich Abdusalomov, Mukhriddin Mukhiddinov, Alpamis Kutlimuratov, Taeg Keun Whangbo

**Affiliations:** Department of Computer Engineering, Gachon University, Sujeong-Gu, Seongnam-si 461-701, Gyeonggi-do, Korea

**Keywords:** fire warning system, smart glasses, blind and visually impaired, YOLOv5, artificial intelligence, flame classification

## Abstract

Early fire detection and notification techniques provide fire prevention and safety information to blind and visually impaired (BVI) people within a short period of time in emergency situations when fires occur in indoor environments. Given its direct impact on human safety and the environment, fire detection is a difficult but crucial problem. To prevent injuries and property damage, advanced technology requires appropriate methods for detecting fires as quickly as possible. In this study, to reduce the loss of human lives and property damage, we introduce the development of the vision-based early flame recognition and notification approach using artificial intelligence for assisting BVI people. The proposed fire alarm control system for indoor buildings can provide accurate information on fire scenes. In our proposed method, all the processes performed manually were automated, and the performance efficiency and quality of fire classification were improved. To perform real-time monitoring and enhance the detection accuracy of indoor fire disasters, the proposed system uses the YOLOv5m model, which is an updated version of the traditional YOLOv5. The experimental results show that the proposed system successfully detected and notified the occurrence of catastrophic fires with high speed and accuracy at any time of day or night, regardless of the shape or size of the fire. Finally, we compared the competitiveness level of our method with that of other conventional fire-detection methods to confirm the seamless classification results achieved using performance evaluation matrices.

## 1. Introduction

The global population is growing rapidly, and a growing number of people are living well into old age, leading to an increase in the number of blind and visually impaired (BVI) people. Elderly and BVI people face a variety of problems while performing routine activities, including housework, environmental awareness, work, traveling, and sports practice, owing to physical, sensory, and cognitive decline. This situation requires considerable attention because the number of people with sight problems is likely to increase tremendously in the coming decades. These numbers are particularly critical in developed countries as advances in healthcare systems have enabled a longer life. Several solutions exist for such problems, and assistive technologies and software are being developed to help BVI people. Employing an early fire detection and notification system for BVI people can reduce property damage, the number of victims, and most importantly, the number of premature deaths.

Fire control has always been a global challenge. According to statistics published by the Korean National Fire Agency, there were 40,030 fires in South Korea in 2019, resulting in 284 fatalities and 2219 injuries. Furthermore, a daily average of 110 fires and 0.8 fire-related fatalities resulted in property damage amounting to KRW 2.2 billion. In 2020, two major Korean cities experienced fires that claimed the lives of over 50 people in each case. In Ulsan, a 33-story tower block building completely burned down, while in Incheon, a warehouse fire occurred [1]. Cooking, heating equipment, electrical distribution and lighting equipment, purposeful fire initiation, and smoking materials are the five factors that contribute to the majority of interior fires and fire fatalities [2]. Cooking was the main contributor to both interior fires and injuries from indoor fires over a five-year period from 2015 to 2019. BVI people are at a greater risk of injury and death owing to fire hazards. Depending on the severity of their vision loss, they may accidentally start a fire during routine household chores and are less likely to eliminate or avoid it. Additionally, a BVI person is highly vulnerable to sustaining burns when attempting to suppress small fires. Some relevant studies on outdoor fire detection have been conducted in the past few years. However, there are insufficient research studies on early fire-detection and notification systems for BVI people using artificial intelligence (AI) approaches in indoor scenes. There are many reasons for this, and our research identified the following problems as the main challenges in early fire-detection studies.

❖ Fire image collection and labeling for indoor fire-detection datasets are challenging owing to the lack of open-access fire image datasets for real-world scenarios. A large amount of labeled training data is key to the success of any deep learning model.
❖ Fires do not have any specified shape or size and even at times, vary in color, hence achieving high accuracy in their detection is a difficult task while the data annotation process is also time-consuming.
❖ Distinguishing between a real fire and an artificial fire or fire-like scene (sunlight and lighting) is also a challenging task, particularly when several errors in real-time fire detection occur in the case of a false alarm. This is because the sunlight and lighting pixel values are very close to the fire color intensities, even though they are not real fires.
❖ Ensuring real-time fire detection and notification along with indoor navigation methods is also a potential challenge because the rapid spread of the fire must not risk the health of the blind and endanger their lives.

Despite these problems, we conducted fire research and developed a new fire-detection and notification system. The proposed fire warning system can immediately detect and notify dangerous fire situations to reduce fire damage to building interiors and to protect lives. The early fire-detection and notification system provides fire prevention and safety information to BVI people in a short period in emergency situations when fires occur in indoor environments.

In summary, the main contributions of this study are as follows:

- Most traditional methods use sensor-based technologies to detect fire scenes [3,4,5,6]; however, these technologies depend on environmental and illumination changes. Further research has shown that camera-based fire-detection systems achieve much better results with high prediction accuracy, low cost, and reduced processing time to enhance fire safety [7,8,9,10].
- AI-based fire detection is a potentially powerful approach to detect flames and to warn building occupants in different indoor environments because it is highly distinctive, does not depend on colors, size, or shape, and is robust to illumination changes [11,12]. However, traditional computer vision or image processing approaches have been applied to simple fires and are appropriate only under certain conditions [13,14,15,16].
- We proposed a fire detection and classification system that can monitor and predict home emergency fire-related situations to provide early warning to BVI people to prevent or reduce the risk to life.
- The use of smart glasses as an assistive technology supports blind users to self-evacuate with minimal assistance during emergencies, such as fires and floods. Furthermore, this assistive technology can be easily adopted in many applications, such as indoor or outdoor navigation, obstacle avoidance, education, and traveling for BVI people.
- The goal of this research is to converge Internet-of-Things devices and AI methods in the field of fire prevention, safety, and indoor navigation for BVI people. This is a unique idea, and smart glasses associated with a security camera can improve the lives of blind users when they perform important daily tasks (such as cooking and heating).

The remainder of this paper is organized as follows. In Section 2, the proposed fire-detection and notification system for BVI residents is described in detail. The experimental findings are described in Section 3. Section 4 concludes the paper and discusses the future directions of the proposed method.

## 2. Proposed Fire-Detection and Notification Method

### 2.1. System Overview

**Figure 1:**
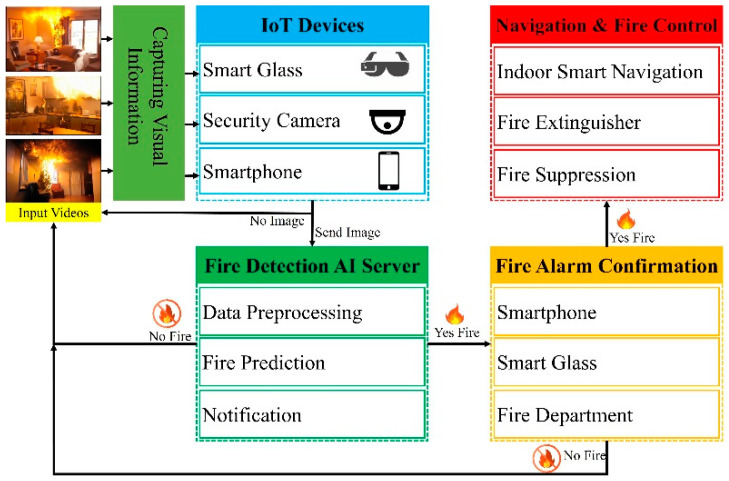
Overall flowchart of the system.

In this subsection, we provide an overview of the proposed method for the rapid and accurate detection of house fires, regardless of their size and shape. Unexpected fires in a house can ignite within a short time and cause unpredictable and frightening consequences. We tested several techniques to achieve our goal and used them successfully in our approach, which is explained in detail in the following subsections. The primary purpose of this research is to develop a new fire-detection method based on AI approaches that can be applied to eliminate fire accidents and an early notification system to avoid unexpected fires and keep BVI people safe. The system is aimed at monitoring home fire hazards to accurately detect even small sparks of flames and notify the BVI occupants and fire department when activated, as illustrated in Figure 1. The YOLOv5 and smart glasses-based approach proposed a fire detection and alerting system that captures images using a small camera and sends them to a server outfitted with an AI module that provides fire detection results with voice feedback. We present a client-server architecture with a smartphone and smart glasses acting as the client and an AI server handling image processing.

It is relatively easy for sighted people to detect fire disasters indoors. However, identifying fire zones and their sizes by BVI people is extremely difficult. One goal of the proposed work is to enable BVI people to deal with the fire while it is very small before the firefighters arrive, or else they can attempt to escape fire regions using a smart navigation system for indoor wayfinding.

### 2.2. Dataset

Dataset collection and generation processes were performed as follows. There are certain specific tasks for which large datasets are unavailable or insufficient, and existing open-access datasets have limited images; these include medical (healthcare) image and fire image datasets [17,18]. AI-based approaches require training data to obtain high prediction accuracy and test data to evaluate the method. To eliminate overfitting, we collected publicly available fire image datasets and Google images for training and testing. However, the training dataset is constrained using this approach for fire detection. To address this, we used picture augmentation techniques and various computer vision techniques to enhance the number of fire frames [9,11].

**Figure 2:**
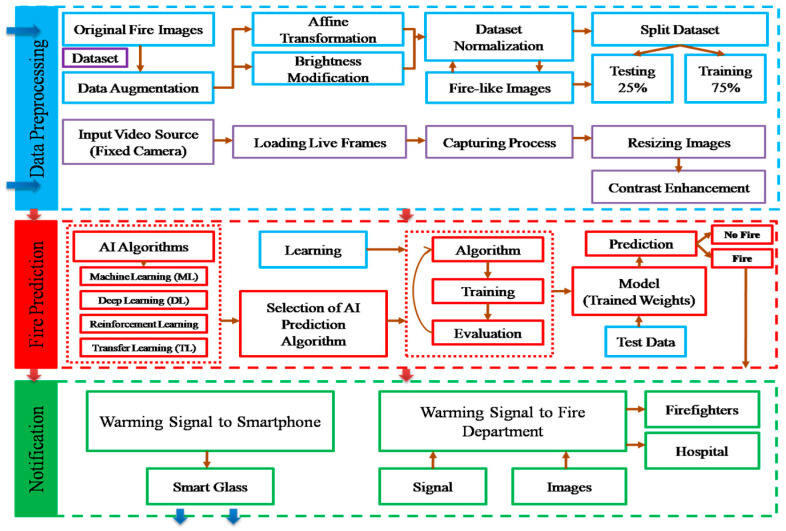
Modular representation of fire detection and classification.

To make the system work in real time, a clear image frame that should be read from a fixed camera every second is delivered to the system. However, the dominant light source is an artificial light source in indoor surroundings, which may decrease the accuracy of fire-detection tasks because of dark scenery and low-quality image of objects. Image contrast enhancement methods have been used to produce high-quality output images in artificial light sources [19,20]. After obtaining suitably enhanced frame sequences, training and testing procedures were performed to create weight files (pretrained weights) to predict possible candidate fire pixels. After gathering sufficient data to overcome the overfitting problem, we used our dataset to train and evaluate the improved YOLOv5 model at the initial stage of our research, as shown in Figure 2.

As illustrated in Figure 2, we divided the fire detection and classification process into three steps. The data processing subsection explains dataset collection and modification with different image enhancement methods. The fire prediction step is mostly focused on selecting an appropriate AI model to apply for fire detection purposes. After training and testing in fire detection cases, the default implementations of the YOLO technique have relatively poor accuracy. We decided to employ and enhance the YOLOv5 network for the effective identification and warning of fire disasters. We recorded the results of quick and accurate fire detection by altering the algorithm.

At the fire notification stage, smart glasses and security cameras record video and capture image frames, which are then sent to the AI server for processing. Following the detection of fire regions, the AI server performs two separate actions for the fire notification stage: (1) it sends voice and text messages to the user’s smartphone, and (2) it sends the fire department a discovered fire image. Regarding the first measure, BVI residents can manage the environment and distinguish between normal household fires and dangerous fires by donning smart glasses. They can self-evacuate if a dangerous fire is confirmed utilizing fire, object, and text recognition techniques, together with object mapping techniques to ascertain the links between various things.

As shown in Table 1, our initial collected fire image dataset consists of 7395 original indoor fire images, which includes fire-like images without fire to decrease false alarm notifications. Existing methods sometimes incorrectly classify fire-like scenarios (environments) as fire hazards because of lamps, sunrise, or sunset and activate the warning system. Therefore, we had to further expand our dataset to include daytime and nighttime non-fire images to eliminate errors.

As mentioned earlier, during the experiments, we found that image data augmentation techniques, such as geometric transformations, brightness/contrast enhancement, and data normalization, proved to be the most effective way to improve the final accuracy rate. The effectiveness of convolutional neural network (CNN) models depends on the size and resolution of the training picture datasets. Therefore, we rotated each original fire image and then flipped each rotated image horizontally to increase the number of images in the fire-detection dataset, as explained in detail in the refs. [9,11,12]. By applying the data augmentation methods to the original 7395 fire images, we increased the total number of images to 90,700. In our previously published papers, we achieved high accuracy using YOLOv3/YOLOv4 networks with images of 608 × 608 and 416 × 416 resolutions. However, the YOLOv5 architecture was suitable for training with an image size of 640 × 640 pixels. If the dataset was sufficiently large and well labeled, we could typically obtain good results without changing the models or training settings. Larger images are typically associated with better outcomes. Each line of the txt file that YOLOv5 requires as annotations for each image should define a bounding box. We trained the model using our own custom dataset, so we needed to perform annotations on the collected images, which is basically labeling the images with their class names so that they can be fed into the model to train it. The image annotations are text files having the same name as that of the image in the following format containing class number, object coordinates, and image height and width. Object coordinates are normalized between 0 and 1 and basically denote the position of the bounded object, the image height and width. We converted the dataset annotations into the well-known PASCAL VOC XML format required by YOLOv5. We divided the entire fire image dataset into training (75%) and test sets (25%).

### 2.3. Fire Detection and Notification

The proposed architecture can be considered as a combination of fire-detection and visually impaired research. After studying AI approaches (machine learning, deep learning, reinforcement learning, and transfer learning) and applying YOLO networks to our research, we significantly improved the home fire-detection and notification performance to eliminate fire fatalities in indoor environments. First, we tested the latest versions of the YOLO algorithms to select the appropriate algorithm and used it in our research for fire detection. Standard versions of the YOLOv5 approach have low accuracy after training and testing in fire-detection situations. After applying computer vision augmentation techniques to the dataset, we observed fast and highly accurate fire disaster detection using YOLOv5.

#### 2.3.1. Model Selection

**Figure 3:**
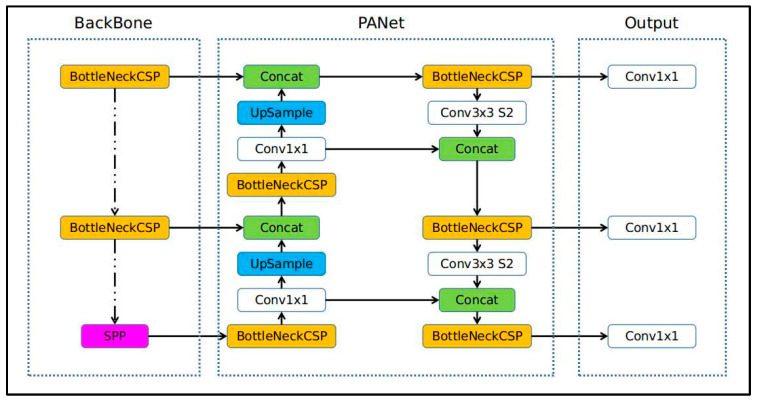
YOLOv5 network structure.

YOLOv5 is a recently introduced CNN that identifies static and dynamic objects with remarkable performance and high accuracy in real time. This model utilizes a single neural network to process the entire image region, then decomposes it into several components, and estimates the candidate bounding boxes and probabilities for each part. YOLOv5 network is a continuous improvement of YOLOv1–YOLOv4 and is divided into three architectures: the backbone based on cross stage partial (CSP) incorporated into Darknet, the neck for boosting information flow based on the path aggregation network (PANet), and the head, which is used to generate YOLO layers for multi-scale prediction [21]. Before being sent to PANet for feature fusion, the data were fed to CSPDarknet for feature extraction. As shown in Figure 3, the YOLO layer delivers detection results by employing three different feature maps (class, score, location, and size).

As shown in Figure 3, the YOLO v5 network is employed as a backbone for extracting important and useful features from the input frame sequences with CSP. The neck model is used by the YOLO v5 network to build feature pyramids that help models generalize successfully with regard to object scaling. This makes it easier to recognize the same thing within a range of scales and sizes. The use of feature pyramids helps models operate efficiently on previously unexplored data. To obtain feature pyramids that enhance the localization of objects at lower levels, YOLOv5 uses PANet as its neck. The final detection stage, which uses three feature maps of sizes 18 × 18, 36 × 36, and 72 × 72, is primarily addressed by the head model. It builds the final output vectors with bounding boxes, objectness scores, and class probabilities using anchor boxes [22,23].

The YOLOv5 family of networks contains five types of models, from the smallest and fastest YOLOv5 nano to YOLOv5 extra-large [24]. YOLOv5n is a newly introduced nano-model that achieves good efficiency for mobile solutions. The compact form, known as YOLOv5s (small model), is appropriate for use with CPU. A medium-sized model, YOLOv5m has 21.2 million parameters. Given that it offers a fair balance between speed and accuracy, it is the model most suited for many datasets and training. The large model of the YOLOv5 family, YOLOv5l, is perfect for datasets in which smaller items need to be found. The largest of the five models, with the highest mean average precision, is YOLOv5x. As shown in Table 2, larger models, such as YOLOv5l and YOLOv5x, are slower to execute and contain more parameters than the others, but they almost always yield superior results.

Based on the size of our dataset and the purpose of our research, we selected YOLOv5m for the implementation in our study. A network that immediately detects and notifies fire accidents is required to reduce fire damage to buildings and protect lives. YOLOv5m has sufficient ability to learn the features of diverse home fire evaluations.

#### 2.3.2. Fire Detection

We focused on moving object detection approaches that work successfully in indoor environments. Because fire regions have the property of movement, they are classified as dynamic objects rather than static objects. When fire detectors recognize fire using selected and improved methods, the monitored alarm system automatically transmits an emergency alarm to the smartphones of BVI people and the fire department to confirm whether it is a human-made fire or fire accident. BVI people may not accurately distinguish daily fires when they wear smart glasses. Therefore, the AI server sends the fire images to the fire department (fire safety office) for verification. If the fire is confirmed by the system operator, they immediately send firefighters to the address where the fire occurred and notify the local hospital.

We introduced a client–server scheme containing a smartphone and smart glasses as the client and an AI server to execute the image analysis [25]. The client section includes smart glasses and a smartphone that transfers information through Bluetooth and a home surveillance camera that records consistently. The AI server simultaneously receives the client’s supply of frames, processes them, and outputs the results in audio format. The built-in speaker or smartphone is used by the smart glasses to connect with users after receiving audio findings. The user uses Bluetooth to link smart glasses to a smartphone. The user can then instruct the smart glasses to capture pictures that are subsequently transmitted to the smartphone. In this scenario, it is possible to decrease the power consumption of glasses, as it is more efficient than continuous video recording. Speech feedback from the AI server is subsequently sent through earphones or speakers. Users of tactile devices can also touch and feel the outline of the prominent object [26]. We used an AI server to perform deep-learning-based computer vision tasks despite the recent introduction of lightweight deep CNN models, because the GPUs in wearable assistive devices are less capable than an AI server. The battery life of smartphones and smart glasses is increased because they are used solely to take pictures.

Moreover, the AI server is practical for enhancing the accuracy of the deep CNN models and introducing new features. The AI server receives images and uses fire-detection and object recognition models to identify fires and objects after text-to-speech [27]. The audio results are then sent to the client as an AI server to respond to their requests. BVI people can hear voice guidelines (“in,” “on,” “next to,” “below,” and “above,” for instance, “fire above oven” or “fire next to chair”) and receive tactile information to assist indoor navigation from fire zones to safe zones [28].

## 3. Experimental Results

We implemented and tested the proposed configuration from the Anaconda 2020 Python distribution on a computer with an 8-core 3.70 GHz CPU, 32 GB RAM, and NVidia GeForce 1080Ti GPUs. As indicated in Table 3, to improve the energy-storage viability of smart glasses and to guarantee real-time system performance, we used a high-performance AI server system.

**Figure 4:**
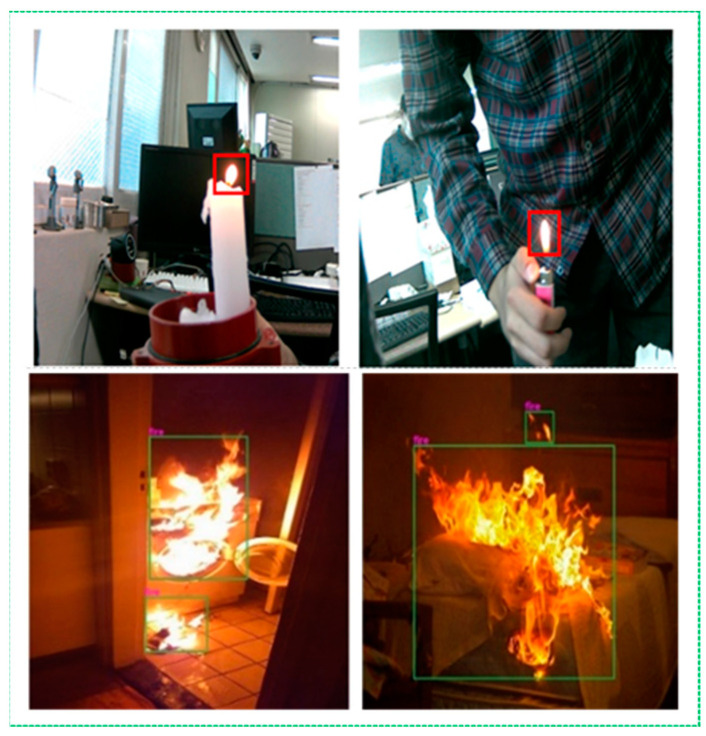
Visible results of the proposed method for small size and multiple flame areas.

**Figure 5:**
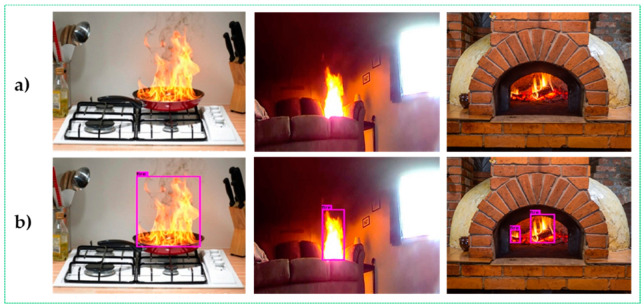
Results of the experiment that may be observed in daytime fire scenarios include the (**a**) input image sequences and the (**b**) output image sequences with fire regions detected.

**Figure 6:**
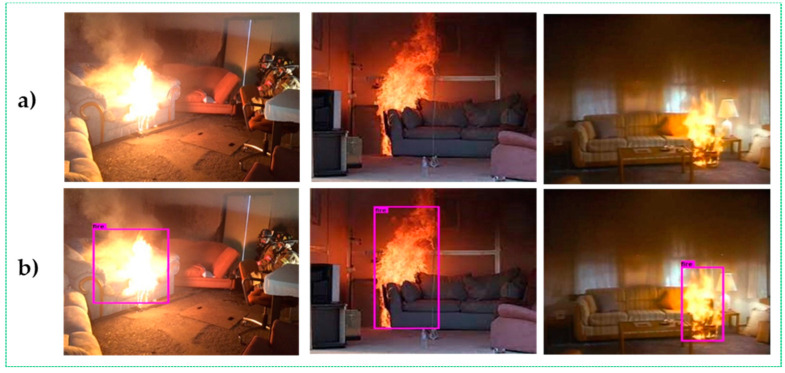
Results of the experiment that may be observed in nighttime fire scenarios include the (**a**) input image sequences and the (**b**) output image sequences with fire regions detected.

In Table 4, the detailed specifications of the smart glasses with a Raspberry Pi 3 Model B+ used to conduct the experiments are listed. In this section, we analyze the application of traditional fire-detection methods and the proposed strategy in terms of qualitative and quantitative outcomes. Examples of visible experiments employing the CSPDarknet-53 feature extractor in daytime and nighttime settings are shown in Figure 4, Figure 5 and Figure 6. DarkNet-53 was used by CSPDarknet-53, a CNN that serves as the backbone for object detection [29,30]. The YOLOv5 network used CSPNet as a backbone to solve repeated gradient data issues and integrate any intensity changes in the feature map.

Owing to its ability to record both full HD 1080p video and high-resolution still images, an 8 MP device camera was employed to capture both images and videos.

### Qualitative Evaluation

Based on the experimental results, it can be concluded that our upgraded fire warning system efficiently recognizes fire regions, even with the same color contrast and similar movements as fire pixels in the background area. Furthermore, our method shows better performance and immediately detects fire accidents, even several fires or small fires in frame sequences, as shown in Figure 4.

We compared well-known fire-detection algorithms to analyze and discuss the quantitative performance of our method. Because the source codes and datasets for these approaches are not publicly available, we used the results in their publications for comparison; however, we are unsure of their veracity. As in our previous studies [31,32,33,34,35], we calculated the F-measure (FM), precision, and recall. The FM score is a weighted average that equalizes the measurements of the recall rates and precision. Precision measures the proportion of successfully anticipated positive observations for all forecasted positive observations. As explained in Equation (1), recall is the proportion of accurately predicted positive observations to all observations in actual class. The average of the FM, recall, and precision of the suggested method was 98.2%. False detection occurred in 1.8% of cases: sometimes, the system classified electrical lamps and blurred spark objects as a real fire. The following equations can be used to calculate the average precision and recall rates of the fire-detection methods:

(1) $$\begin{array}{c} \mathrm{Precision}=\frac{\mathrm{TP}}{\mathrm{TP}+\mathrm{FP}},\\ \mathrm{Recall}=\frac{\mathrm{TP}}{\mathrm{TP}+\mathrm{FN}}, \end{array}$$

where TP stands for “true positives,” FP for “false positives,” and FN for “false negatives,” all of which indicate correctly discovered fire zones. Precision was calculated as the ratio of true positives to both true and false positives. The proportion of genuine positives to both true positives and false negatives is known as the recall.

Then, using (2) FM is calculated as follows

(2) $$\mathrm{FM}=\frac{2\times \mathrm{precision}\times \mathrm{recall}}{\mathrm{precision}+\mathrm{recall}}.$$

Actual applications may result in extremely dark, hazy, or obstructed fire areas or pixels in photographs of natural scenes (by cloud). Additionally, we evaluated the effectiveness of the fire-detection techniques using the Jaccard index or the intersection-over-union (IoU) measure, which expresses the ratio of items that the two sets share to the total number of the objects. It is defined as the area of union of the detected fire region and the ground truth divided by the area of overlap of the detected fire region and the ground truth. In other words, it is a useful statistic for evaluating detection outcomes (3).

(3) $$\mathrm{IoU}=\frac{\mathrm{groundTruth}\cap \mathrm{prediction}}{\mathrm{groundTruth}\cup \mathrm{prediction}}$$

The FM score and IoU values range from 0 to 1, with 1 representing the optimal value for these metrics. Table 5 provides an analysis of our strategy in comparison with other previously reported fire-detection techniques.

**Figure 7:**
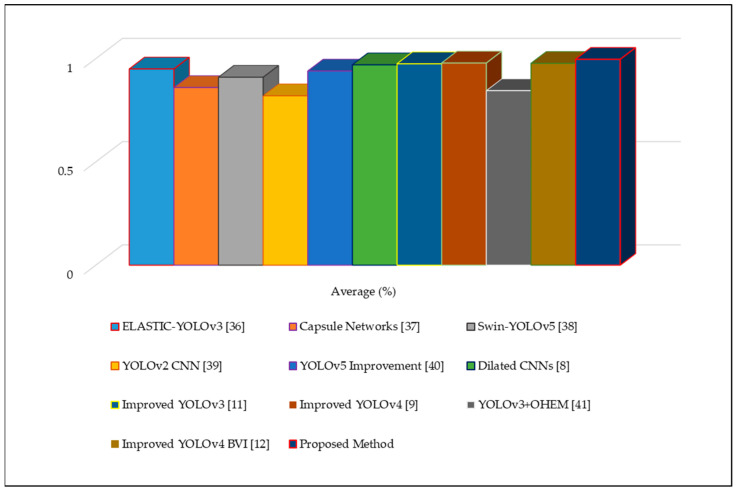
Quantitative results of speech signal feature extraction approaches using vertical graphs.

Furthermore, based on Table 5, we performed a statistical analysis to determine the average accuracy of the examination methods using the average evaluation metrics, as shown in Figure 7. The improved fire warning system yielded an approximately 98.2% accuracy, whereas other approaches yielded accuracies approximately between 84% to 97%. We used the results of previous studies for comparison; however, the accuracy of these values is not easily verifiable because the source codes and datasets of these methods are not publicly available to confirm their real performance. Nevertheless, in the case of standard scenes, the proposed method experimentally proved its ability to provide excellent fire-detection accuracy by reducing the computational time, even when the fire region was small or there were multiple fire regions.

**Figure 8:**
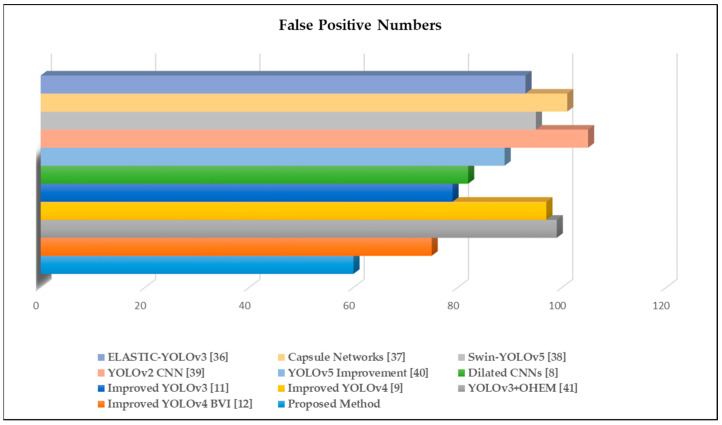
Visible results of false-positive speech signal feature extraction experiments.

Additionally, we assessed the false-positive records of the selected methodologies, as illustrated in Figure 8. The trials revealed that the suggested technique contained the least errors compared with the weight files in the existing algorithms after adding fire-like and small-size fire photos to the dataset.

In addition, we obtained the processing time performance for each phase of the proposed system, including the time required for image processing for the fire detection and notification module in the AI server, 5G/Wi-Fi image transmission between the smartphone and server, and Bluetooth image transmission between the smart glasses and smartphone. Table 6 displays the typical processing time for each stage. The total time at every stage is 1.26 s, which is allowed under actual conditions.

We summarize the robustness of our published papers using the proposed method in different categories using quantitative and qualitative performance results, as shown in Table 7. Based on the evaluated scores, the performance of the proposed approach did not suffer when small fire areas were included and successfully distinguished artificial or non-fire scenes (sunsets, sunshine, lighting, and electric lamps) from real fires. In an indoor environment, the best results, with enhanced detection time and accuracy, were obtained for solving early fire classification and notification challenges by applying the proposed method.

The outcomes of fire identification and notification techniques can be divided into three categories: robust, standard, and powerless. Robust measures show that the method is applicable to all types of fires. The algorithm may fail in some circumstances, such as when it occurs frequently or the fire spreads, according to normal standards. Powerless evidence suggests that algorithms are unreliable in the presence of noise or color, and the fire identification procedure frequently modifies the initial geometry of moving fires. Spread fire elimination shows that image-based fire detectors can detect fires in many directions.

## 4. Conclusions and Future Directions

In this study, we developed improved smart fire warning systems based on advanced technologies to enhance firefighting safety and life protection. The proposed AI-based fire-detection method can be used in various environments, such as smart/safe cities, and for monitoring fires in urban areas to protect visually impaired people. In our study, we focused on its application in early fire-detection systems based on cameras and wireless systems. These systems are becoming more attractive for use in housing, as they accommodate patients with impairments and disabilities who live alone because they are perceived to be more secure from dangerous fire scenes. Similarly, we expect that the proposed system can be effectively used in fire safety industries. The system detects and notifies catastrophic fire outbreaks in real time with high speed and accuracy. Early detection of a fire accelerates the process of eliminating it; thus, the fire poses a lesser threat to the health and lives of people including the firefighters. 

Another major contribution of the proposed system is that it can analyze the intentions of humans as well as the safety of generated fires through artificial intelligence (AI) and Internet-of-Things (IoT) approaches. Real-time fire detectors can make rapid decisions during emergencies and ignore alarms when cameras detect fires created by humans. Thus, this AI-based fire-detection approach helps prevent the occurrence of false alarms. Furthermore, we generated a large fire image dataset that contained 7395 fire and non-fire images for model training and testing. To effectively identify the target data and overcome overfitting issues, deep CNNs learn critical features from big datasets. By contrasting the proposed system with other well-known one-stage object detectors, we assessed the qualitative and quantitative performance of the system during the experiments. The experimental findings and evaluation demonstrated that the enhanced YOLOv5 model produced seamless classification results while performing slightly better accuracy using our fire detection dataset.

Finally, we hope that the proposed system will be effective in various medical centers focused on blindness and visual impairments to control and preserve the health and lives of patients from unpredictable fire accidents. Future tasks include solving blurry problems under dark conditions and increasing the accuracy of the approach. We plan to develop a small model with reliable fire detection performance using 3D CNN and 3D U-Net in the IoT Environment [42,43,44].

## Figures and Tables

**Table 1:** Dataset distribution for fire classification research.

Dataset	Flame Frames				Non-Flame Frames				Total
	GitHub	Kaggle	Flickr	Google	GitHub	Kaggle	Flickr	Google	
Indoor environment	1572	1693	855	741	962	987	327	258	7395

**Table 2:** Description of the YOLOv5 models.

Models	Backbone	Image Size	AP	AP_50_	Speed_ms_	FLOP_S_	Param_M_	Epochs
YOLOv5n	CSPDarknet-53	640 × 640	28.0%	45.8%	1.7	4.5	1.9	
YOLOv5s	CSPDarknet-53		37.4%	57.6%	2.1	16.5	7.2	
YOLOv5m	CSPDarknet-53		45.4%	63.9%	2.8	49.1	21.2	300
YOLOv5l	CSPDarknet-53		49.1%	65.5%	3.7	110	46.5	
YOLOv5x	CSPDarknet-53		50.6%	67.4%	5.9	205.7	86.7	

**Table 3:** AI server specifications.

Hardware	Detailed Specifications
Graphic Processing Unit	GeForce RTX 2080 TI 11 GB (2 are installed)
Central Processing Unit	Intel Core 9 Gen i7-9700k (4.90 GHz)
Random Access Memory	DDR4 16 GB (4 are installed)
Storage	SSD: 512 GB HDD: 2 TB (2 are installed)
Motherboard	ASUS PRIME Z390-A
Operating System	Ubuntu Desktop
Local Area Network	Internal port—10/100 Mbps External port—10/100 Mbps
Power	1000 W (+12 V Single Rail)

**Table 4:** Full description of the smart glasses.

Hardware	Detailed Specifications
Processor	Broadcom BCM2837B0 chipset, 1.4 GHz Quad-Core ARM Cortex-A53 (64 Bit)
Graphic Processing Unit	Dual Core Video Core IV^®^ Multimedia Co-Processor
Memory	1 GB LPDDR2 SDRAM
Connectivity Wireless LAN	2.4 GHz and 5 GHz IEEE 802.11.b/g/n/ac, maximum range of 100 m
Connectivity Bluetooth	IEEE 802.15 Bluetooth 4.2, BLE, maximum range of 50 m
Connectivity Ethernet	Gigabit Ethernet over USB 2.0 (maximum throughput 300 Mbps)
Video and Audio Output	1 × full size HDMI, Audio Output 3.5 mm jack, 4 × USB 2.0 ports
Camera	15-pin MIPI Camera Serial Interface (CSI-2)
Operating System	Boots from Micro SD card, running a version of the Linux operating system or Windows 10 IoT
SD Card Support	Micro SD format for loading operating system and data storage
Power	5 V/2.5 A DC via micro-USB connector

**Table 5:** Quantitative results of fire identification and notification approaches.

Algorithms	P (%)	R (%)	FM (%)	IoU (%)	Average (%)
ELASTIC-YOLOv3 [36]	0.956	0.969	0.937	0.901	0.940
Capsule Networks [37]	0.864	0.816	0.892	0.922	0.873
Swin-YOLOv5 [38]	0.948	0.936	0.942	0.958	0.943
YOLOv2 CNN [39]	0.834	0.716	0.762	0.882	0.801
YOLOv5 Improvement [40]	0.937	0.942	0.943	0.941	0.940
Dilated CNNs [8]	0.971	0.974	0.982	0.957	0.971
Improved YOLOv3 [11]	0.968	0.982	0.985	0.967	0.975
Improved YOLOv4 [9]	0.976	0.958	0.979	0.982	0.979
YOLOv3 + OHEM [41]	0.866	0.778	0.892	0.863	0.845
Improved YOLOv4 BVI [12]	0.968	0.981	0.974	0.974	0.977
Proposed Method	0.982	0.997	0.997	0.991	0.982

**Table 6:** Average frame processing time (in seconds) for each sequence.

Transmission and Image Processing	Average Frame Processing Time (s)
Bluetooth transmission	0.11
5G/Wi-Fi transmission	0.32
Fire detection and notification	0.83
Total	1.26

**Table 7:** Evaluation of the effectiveness of fire detection using different characteristics.

Criterion	Improved YOLOv3 [11]	Improved YOLOv4 [9]	Improved YOLOv4 BVI [12]	Proposed Method
Scene Independence	standard	robust	standard	robust
Object Independence	standard	robust	robust	standard
Robust to Noise	powerless	robust	standard	robust
Robust to Color	standard	standard	powerless	robust
Small Fire Detection	robust	standard	robust	robust
Multiple Fire Identification	standard	powerless	powerless	robust
Processing Time	powerless	standard	robust	robust

## Data Availability

Data sharing not applicable.
